# Burden of Rifampicin Resistance Among Patients With Tuberculosis at a Tertiary Care Center in Uttarakhand, India: A Prospective Cross-Sectional Study

**DOI:** 10.7759/cureus.114330

**Published:** 2026-08-11

**Authors:** Saurabh Negi, Malvika Singh, Iva Chandola, Jagdish Rawat

**Affiliations:** 1 Microbiology, Shri Guru Ram Rai Institute of Medical and Health Sciences, Dehradun, IND; 2 Microbiology, Himalayan Institute of Medical Sciences, Dehradun, IND; 3 Pulmonology, Shri Guru Ram Rai Institute of Medical and Health Sciences, Dehradun, IND

**Keywords:** multidrug-resistant tuberculosis, mycobacterium tuberculosis, polymerase chain reaction, rifampicin resistance, rpob gene, tuberculosis

## Abstract

Introduction

Tuberculosis (TB) remains a major global health problem, and the emergence of drug-resistant *Mycobacterium tuberculosis* poses a significant challenge to disease control. Rifampicin resistance, which is predominantly associated with a mutation in the *rpoB* gene, serves as a key marker for multidrug-resistant tuberculosis (MDR-TB). Early molecular detection of rifampicin resistance is essential for timely initiation of appropriate therapy and prevention of disease transmission.

Objective

To determine the prevalence of rpoB gene mutation associated with rifampicin resistance among patients with tuberculosis and to evaluate associated demographic and clinical risk factors in a tertiary care hospital setting.

Materials and methods

This prospective cross-sectional study was conducted over 18 months (July 2024-January 2026) in the Departments of Microbiology and Pulmonary Medicine at SGRR Institute of Medical and Health Sciences, Dehradun. Seventy patients with microbiologically confirmed tuberculosis were enrolled. Clinical specimens were processed using the N-acetyl-L-cysteine-sodium hydroxide method. Detection of Mycobacterium tuberculosis was performed using the RT-PCR technique with Truenat kit (3B BlackBio Dx, India). DNA extracted using the QIAamp DNA Mini Kit was subjected to allele-specific polymerase chain reaction (PCR) targeting a 531 specific codon of the rpoB gene. Amplified products were analyzed by agarose gel electrophoresis. Statistical analysis was performed using IBM SPSS Statistics version 27.0 (IBM Corp., Armonk, NY, USA), and a p-value <0.05 was considered statistically significant.

Results

The majority of patients belonged to the 31-45 years age group (24, 34.3%), and males constituted 45 (64.3%) of the study population. Pulmonary tuberculosis accounted for 98.6% of cases. rpoB gene mutations indicative of rifampicin resistance were detected in 5 of 70 patients, yielding a prevalence of 7.1%. All mutation-positive cases were observed among pulmonary TB patients. Although higher proportions of rpoB mutation were noted among previously treated patients (3, 21.4%) (n=14), those with treatment default history (2, 40.0%) (n=5), patients with cavitary disease on chest radiography (4, 21.1%) (n=19), HIV co-infected individuals (1, 16.7%) (n=6), and diabetic patients (2, 11.1%) (n=18). However, none of these associations was statistically significant (p>0.05).

Conclusion

Rifampicin resistance mediated by rpoB gene mutation was identified in 7.1% of tuberculosis patients in the present study. Higher frequencies among previously treated patients and those with cavitary disease were observed; however, no statistically significant associations with the evaluated risk factors could be deduced. Early molecular detection of rifampicin resistance and focused screening of high-risk groups are crucial for strengthening tuberculosis control and improving clinical outcomes.

## Introduction

Tuberculosis (TB) is a major global public health concern and is one of the most significant infectious diseases contributing to illness and death worldwide. It affects people of all ages, sexes and geographic regions. According to global estimates, around 10 million individuals developed TB in 2020, resulting in approximately 1.5 million deaths [1]. In 2021, the disease burden increased further, with an estimated 10.6 million new cases and about 1.6 million deaths, reinforcing the impact of TB and the need for sustained control efforts [2].

India carries a substantial proportion of the global tuberculosis burden and contributes nearly 28% of the cases reported among high-burden countries in the South-East Asia region. Findings from the National TB Prevalence Survey, 2021 indicated that approximately 31.3% of individuals aged 15 years and above had evidence of tuberculosis infection [3]. Although considerable progress has been achieved through the National TB Elimination Programme (NTEP), tuberculosis continues to be a significant public health challenge in the country.

The development of drug-resistant strains of Mycobacterium tuberculosis poses a major obstacle to tuberculosis control. In India, multidrug-resistant tuberculosis (MDR-TB), characterized by resistance to both isoniazid and rifampicin, has been identified in approximately 3.9% of newly diagnosed cases and 13.4% of previously treated patients [4]. Resistance to rifampicin is widely regarded as a reliable surrogate marker for identifying multidrug-resistant tuberculosis (MDR-TB) [5].

Culture-based drug susceptibility testing remains the gold standard for assessing drug resistance. However, the slow growth rate of Mycobacterium tuberculosis results in a prolonged turnaround time of several weeks [6]. Delay in diagnosis may contribute to inappropriate treatment and continued transmission of infection. Rapid molecular diagnostic methods, such as polymerase chain reaction (PCR) and cartridge-based nucleic acid amplification test (CBNAAT), have emerged as valuable tools for the early detection of tuberculosis and drug resistance, offering high sensitivity and specificity [7, 8].

Due to the limitations associated with conventional diagnostic approaches, increasing emphasis has been placed on the adoption of molecular techniques for the diagnosis of tuberculosis. As part of the National Strategic Plan (2020-2025), the Government of India has promoted rapid molecular diagnostics through initiatives such as the TB Mukt Bharat Abhiyaan, which has contributed to an improvement in diagnostic sensitivity from around 65% with smear microscopy to nearly 85% [9].

In this context, the present study was undertaken in alignment with the objectives of the National TB Elimination Programme to determine the burden of multidrug-resistant Mycobacterium tuberculosis in a tertiary care hospital setting using conventional PCR-based detection of rpoB gene mutation.

## Materials and methods

This hospital-based prospective cross-sectional study was conducted in the Departments of Microbiology and Pulmonary Medicine at SGRR Institute of Medical and Health Sciences, Dehradun, over a period of 18 months (July 2024 to January 2026).

Inclusion criteria

The study included patients from all age groups and both genders with confirmed Mycobacterium tuberculosis (MTB) infection diagnosed by molecular technique, RT-PCR using the TrueNat kit (3B BlackBio Dx, India), who gave informed consent.

Exclusion criteria

Patients already receiving anti-tubercular therapy, patients with inadequate or improperly collected samples and patients unwilling to participate were excluded from the study.

Sample size

The sample size was calculated using the single population proportion formula, n=Z2p(1−p)/d2. An anticipated prevalence of multidrug/rifampicin-resistant tuberculosis (MDR/RR-TB) of 3.5% was considered based on the World Health Organization Global Tuberculosis Report 2021 [1]. At a 95% confidence level (Z = 1.96) and an absolute allowable error of 5% (d = 0.05), the minimum required sample size was calculated as 52. A total of 70 eligible patients were enrolled during the study period, exceeding the minimum calculated sample size.

Clinical samples, predominantly sputum, along with bronchoalveolar lavage (BAL) and pus, were collected under aseptic conditions following standard biosafety guidelines. Samples were transported to the molecular laboratory under recommended conditions.

Sample decontamination and processing

Clinical samples, including sputum, bronchoalveolar lavage (BAL), and pus, were processed using manufacturer guidelines for RT PCR.

The decontaminated samples were subjected to DNA extraction using the QIAamp DNA Mini Kit (QIAGEN, Germany) according to the manufacturer’s instructions. Extracted DNA was eluted in buffer AE and stored under appropriate conditions until further molecular analysis.

Molecular detection of MTB was performed using RT-PCR. Allele-specific PCR amplification of positive samples was carried out using in-house protocols. Amplification of a 315 bp fragment of the rpoB gene, targeting codon 531-specific mutation, was performed using specific primers:

Forward: 5’-CACAAGCGCCGACTGTC-3’

Reverse: 5’-TTGACCCGCGCGTACAC-3’

Gel electrophoresis

PCR products were analyzed by 2% agarose gel electrophoresis using a 100 bp DNA ladder as the molecular size marker. Amplified products were visualized under UV illumination using a gel documentation system. Samples showing amplification only with the wild-type primer were interpreted as lacking the targeted codon 531 mutation. Positive and negative controls were included in each PCR run to ensure the validity and reliability of the assay.

Quality control

Internal validity was ensured by using standardized molecular techniques and calibrated instruments. All procedures were conducted following strict standard operating protocols. Reliability was maintained through adherence to uniform procedures and independent verification of results by trained personnel.

Ethical considerations

The study was approved by the Institutional Ethics Committee. Written informed consent was obtained from all participants. Confidentiality of patient data was strictly maintained throughout the study.

## Results

As shown in Table 1, the highest proportion of participants belonged to the 31-45-year age group (34.3%), followed by the 46-60-year (27.1%) and 18-30-year (25.7%) age groups, while participants aged >60 years constituted 12.9% of the study population. The distribution of participants across the age groups did not differ significantly from an equal expected distribution (χ² = 6.686, df = 3, p = 0.083).

**Table 1:** Age distribution of participants (n=70) p-value calculated using the chi-square goodness-of-fit test, assuming an equal expected distribution across age groups.

Age Group (Years)	Number of Participants (n)	Percentage (%)	χ²	p-value*
18–30	18	25.7		
31–45	24	34.3		
46–60	19	27.1		
>60	9	12.9	6.686	0.083
Total	70	100		

As shown in Table 2, males constituted the majority of study participants (45, 64.3%), while females accounted for 25 (35.7%), indicating a male predominance in the study population.

**Table 2:** Gender distribution of the study participants (n = 70) p-value calculated using the chi-square goodness-of-fit test.

Gender	Frequency (n)	Percentage (%)	χ²	p-value*
Male	45	64.3		
Female	25	35.7		
Total	70	100	5.714	0.017

The distribution of tuberculosis cases is presented in Table 3. Of the total 70 cases, 69 (98.6%) were pulmonary tuberculosis, whereas only 1 case (1.4%) was extrapulmonary tuberculosis, demonstrating a marked predominance of pulmonary involvement.

**Table 3:** Distribution of cases based on pulmonary and extrapulmonary manifestations (n=70) Using a chi-square goodness-of-fit test, there was a highly significant predominance of pulmonary tuberculosis compared with extrapulmonary tuberculosis (98.6% vs. 1.4%; χ² = 66.06, p < 0.001).

Type of TB	Number (n = 70)	Percentage (%)	χ²	p-value*
Pulmonary TB	69	98.6	66.06	<0.001
Extrapulmonary TB	1	1.4		
Total	70	100.0		

The association between type of tuberculosis and rpoB mutation is depicted in Table 4. Among pulmonary TB cases, 5 out of 69 patients (7.2%) were positive for Rifampicin resistance.

**Table 4:** Association between type of tuberculosis and rpoB mutation among MTB-positive patients (n = 70) Data are presented as n (%). Fisher's exact test was used to assess the association between the type of tuberculosis and rifampicin resistance because of the small expected cell frequencies. A p-value <0.05 was considered statistically significant. No statistically significant association was observed (p = 1.000).

Type of TB	rpoB mutation Positive, n (%)	rpoB mutation Negative, n (%)	Total, n (%)	χ²	p-value*
Pulmonary TB	5 (7.2)	64 (92.8)	69 (98.6)	0.078	1.000
Extrapulmonary TB	0 (0.0)	1 (100.0)	1 (1.4)		
Total	5 (7.1)	65 (92.9)	70 (100.0)		

Table 5 presents the distribution of the rpoB gene mutation according to selected demographic and clinical risk factors among MTB-positive patients. Treatment default history (OR = 12.62, 95% CI: 0.79-169.92; p = 0.038) and cavitary disease on chest radiography (OR = 12.74, 95% CI: 1.15-669.22; p = 0.017) were significantly associated with the presence of the rpoB mutation. Previous tuberculosis treatment showed a borderline association (OR = 7.06, 95% CI: 0.72-93.94; p = 0.051), whereas HIV co-infection, diabetes mellitus, and age >45 years were not significantly associated with the mutation (p > 0.05).

**Table 5:** Distribution of mutation in rpoB gene according to selected demographic and clinical risk factors among MTB-positive patients (n = 70) Associations between selected demographic and clinical risk factors and rpoB mutation were evaluated using the two-sided Fisher's exact test. Exact odds ratios (ORs) with 95% exact confidence intervals (CIs) were calculated using the conditional maximum likelihood method. A p-value < 0.05 was considered statistically significant. CXR: Chest X-Ray; MTB: Mycobacterium tuberculosis

Risk Factor	rpoB+ n (%)	rpoB− n (%)	Exact Odds Ratio (95% Exact CI)	P value (Fisher's exact, two-sided)
Previous TB treatment (n = 14)	3 (21.4)	11 (78.6)	7.06 (0.72–93.94)	0.051
Treatment default history (n = 5)	2 (40.0)	3 (60.0)	12.62 (0.79–169.92)	0.038
HIV co-infection (n = 6)	1 (16.7)	5 (83.3)	2.93 (0.05–38.98)	0.370
Diabetes mellitus (n = 18)	2 (11.1)	16 (88.9)	2.02 (0.16–19.31)	0.597
Cavitary disease on CXR (n = 19)	4 (21.1)	15 (78.9)	12.74 (1.15–669.22)	0.017*
Age >45 years (n = 28)	3 (10.7)	25 (89.3)	2.37 (0.25–30.22)	0.383

## Discussion

In the current study, the largest proportion of patients was observed in the 31-45 years age group (34.3%). The concentration of cases among economically active adults is also reported in previous reports. Sailo et al. demonstrated that rifampicin-resistant tuberculosis was most frequently encountered among patients aged 25-34 years [10], while Sharew et al. reported the highest prevalence of tuberculosis among individuals in the 15-29 years age group [11].

Although a considerable number of patients in the present study were between 18 and 30 years of age, the highest frequency of cases was recorded in the 31-45 years category. The relatively older age distribution observed may be due to factors such as delays in diagnosis, late presentation to healthcare facilities, and the referral pattern associated with tertiary care centers. Differences in age-specific distribution between studies may also arise from variations in local disease epidemiology, healthcare accessibility, and patterns of transmission. Collectively, these findings suggest that tuberculosis predominantly affects individuals in the productive years of life, thereby posing a significant socioeconomic burden.

Male patients constituted 64.3% of the study population, a finding similar to that reported by Torokaa et al., who observed that 69.1% of cases in a GeneXpert-based study were males. The higher burden among men has been widely described in tuberculosis epidemiology. This pattern may be related to greater occupational exposure, migration and crowded living or working conditions, which favor transmission of Mycobacterium tuberculosis [12-14]. Furthermore, lifestyle factors such as tobacco use and alcohol consumption, which are more common among males in many populations, may increase the risk of infection and disease progression by compromising pulmonary immune defenses [15].

Pulmonary tuberculosis accounted for the majority (98.6%) of cases in the present study, while extrapulmonary tuberculosis (EPTB) comprised only 1.4%. This predominance is attributable to the respiratory specimen-based study design, with sputum and bronchoalveolar lavage forming the majority of samples. These specimens typically have a higher bacillary load, resulting in better diagnostic performance of CBNAAT and other molecular assays [6]. In contrast, EPTB is often paucibacillary and frequently requires invasive sampling and a combination of clinical, radiological, and histopathological evidence for diagnosis, which may limit microbiological confirmation and contribute to its lower representation in laboratory-based studies [16,17]. Similar findings have been reported by Sharew et al., whose analysis was restricted to sputum specimens, and by Miotto et al., who also observed a predominance of respiratory samples in studies evaluating drug resistance [11,18].

Limitations

This study is limited by its small sample size and low number of mutation-positive cases, which reduce statistical power. Being a single-center, tertiary care-based study, the findings may not be generalizable. The predominance of pulmonary cases limits conclusions on extrapulmonary tuberculosis, and the absence of confirmatory sequencing or phenotypic drug susceptibility testing restricts validation of rpoB mutation findings. Also, only a single target region of the rpoB gene was analyzed for mutation detection, which may have resulted in missing mutations located outside the targeted sequence.

## Conclusions

The present study demonstrated that tuberculosis predominantly affected individuals in the economically productive age group, with a clear male predominance and a majority of pulmonary cases. Rifampicin resistance, indicated by rpoB gene mutation, was detected in 7.1% of patients, highlighting a moderate burden of drug resistance in the study population. Although no statistically significant associations were observed between rpoB mutation and the evaluated risk factors, higher proportions were noted among previously treated patients, those with treatment default, suggesting clinically relevant trends. These findings cannot ignore the importance of early molecular detection of rifampicin resistance and targeted screening of high-risk groups to improve tuberculosis control and treatment outcomes. Also, future multicentric studies with larger sample sizes and comprehensive sequencing of the rpoB gene are required to overcome these limitations and provide a more representative understanding of rifampicin resistance-associated mutations.
